# Supplementary material for: Construction and validation of a nomogram model to predict the poor prognosis in patients with pulmonary cryptococcosis
Source: PeerJ. 2024 Mar 11;12:e17030. doi: 10.7717/peerj.17030 (PMC10939030; doi:10.7717/peerj.17030)
Supplement: Table S1 [file peerj-12-17030-s001.docx]

**Table S1.** Comparison of dichotomous variables between all patients with and without poor prognosis.

|  |  | Poor prognosis | Improved prognosis | χ^2^ | P |
| --- | --- | --- | --- | --- | --- |
| Gender | Male | 258(77.5%) | 75(22.5%) | 0.203 | 0.652 |
|  | Female | 152(79.2%) | 40(20.8%) |  |  |
| Onset season | Spring | 95(72%) | 37(28%) | 5.104 | 0.164 |
|  | Summer | 102(79.7%) | 26(20.3%) |  |  |
|  | Autumn | 104(77.6%) | 30(22.4%) |  |  |
|  | Winter | 109(83.2%) | 22(16.8%) |  |  |
| Other lung infections | No | 325(80.6%) | 78(19.4%) | 6.592 | 0.01 |
|  | Yes | 85(69.7%) | 37(30.3%) |  |  |
| Fever | Without | 212(79.7%) | 54(20.3%) | 0.811 | 0.368 |
|  | Have | 198(76.4%) | 61(23.6%) |  |  |
| Headache | Without | 231(78%) | 65(22%) | 0.001 | 0.973 |
|  | Have | 179(78.2%) | 50(21.8%) |  |  |
| Twitch | Without | 394(78.3%) | 109(21.7%) | 0.387 | 0.534 |
|  | Have | 16(72.7%) | 6(27.3%) |  |  |
| Nausea | Without | 302(78.6%) | 82(21.4%) | 0.253 | 0.615 |
|  | Have | 108(76.6%) | 33(23.4%) |  |  |
| Cough | Without | 243(77.4%) | 71(22.6%) | 0.228 | 0.633 |
|  | Have | 167(79.1%) | 44(20.9%) |  |  |
| Shortness of breath | Without | 375(79.4%) | 97(20.6%) | 5.01 | 0.025 |
|  | Have | 35(66%) | 18(34%) |  |  |
| Mental symptoms | Without | 374(79.6%) | 96(20.4%) | 5.739 | 0.017 |
|  | Have | 36(65.5%) | 19(34.5%) |  |  |
| Asymptomatic | No | 347(76.6%) | 106(23.4%) | 4.314 | 0.038 |
|  | Yes | 63(87.5%) | 9(12.5%) |  |  |
| Imaging | No lesion | 39(75%) | 13(25%) | 3 | 0.223 |
|  | Limitations | 205(81.3%) | 47(18.7%) |  |  |
|  | Diffuse | 166(75.1%) | 55(24.9%) |  |  |
| Diabetes | Without | 324(77.5%) | 94(22.5%) | 0.408 | 0.523 |
|  | Have | 86(80.4%) | 21(19.6%) |  |  |
| Tuberculosis | Without | 360(77.6%) | 104(22.4%) | 0.605 | 0.437 |
|  | Have | 50(82%) | 11(18%) |  |  |
| Malignant tumor | Without | 383(77.5%) | 111(22.5%) | 1.561 | 0.212 |
|  | Have | 27(87.1%) | 4(12.9%) |  |  |
| Hematological malignancies | Without | 395(78.2%) | 110(21.8%) | 0.116 | 0.733 |
|  | Have | 15(75%) | 5(25%) |  |  |
| Organ transplant | Without | 386(78.1%) | 108(21.9%) | 0.009 | 0.925 |
|  | Have | 24(77.4%) | 7(22.6%) |  |  |
| Other comorbidities | Without | 254(78.4%) | 70(21.6%) | 0.044 | 0.833 |
|  | Have | 156(77.6%) | 45(22.4%) |  |  |
| HIV | Without | 337(78.6%) | 92(21.4%) | 0.29 | 0.59 |
|  | Have | 73(76%) | 23(24%) |  |  |
| Capsular antigen | (-) | 146(76%) | 46(24%) | 0.746 | 0.388 |
|  | (+) | 264(79.3%) | 69(20.7%) |  |  |
| Hormone Therapy | No | 341(78.6%) | 93(21.4%) | 0.332 | 0.565 |
|  | Yes | 69(75.8%) | 22(24.2%) |  |  |
| Immunosuppressant | Unused | 352(76.9%) | 106(23.1%) | 3.222 | 0.073 |
|  | Use | 58(86.6%) | 9(13.4%) |  |  |
| Cytotoxic drugs | Unused | 388(77.9%) | 110(22.1%) | 0.191 | 0.662 |
|  | Use | 22(81.5%) | 5(18.5%) |  |  |
